# High photocatalytic activity of Fe_2_O_3_/TiO_2_ nanocomposites prepared by photodeposition for degradation of 2,4-dichlorophenoxyacetic acid

**DOI:** 10.3762/bjnano.8.93

**Published:** 2017-04-24

**Authors:** Shu Chin Lee, Hendrik O Lintang, Leny Yuliati

**Affiliations:** 1 Centre for Sustainable Nanomaterials, Ibnu Sina Institute for Scientific and Industrial Research, Universiti Teknologi Malaysia, 81310 UTM Johor Bahru, Johor, Malaysia; 2 Ma Chung Research Center for Photosynthetic Pigments, Universitas Ma Chung, Villa Puncak Tidar N-01, Malang 65151, East Java, Indonesia

**Keywords:** 2,4-dichlorophenoxyacetic acid, Fe_2_O_3_/TiO_2_, herbicide degradation, heterojunction, holes and superoxide radicals, photocatalyst, photodeposition, water purification

## Abstract

Two series of Fe_2_O_3_/TiO_2_ samples were prepared via impregnation and photodeposition methods. The effect of preparation method on the properties and performance of Fe_2_O_3_/TiO_2_ for photocatalytic degradation of 2,4-dichlorophenoxyacetic acid (2,4-D) under UV light irradiation was examined. The Fe_2_O_3_/TiO_2_ nanocomposites prepared by impregnation showed lower activity than the unmodified TiO_2_, mainly due to lower specific surface area caused by heat treatment. On the other hand, the Fe_2_O_3_/TiO_2_ nanocomposites prepared by photodeposition showed higher photocatalytic activity than the unmodified TiO_2_. Three times higher photocatalytic activity was obtained on the best photocatalyst, Fe_2_O_3_(0.5)/TiO_2_. The improved activity of TiO_2_ after photodeposition of Fe_2_O_3_ was contributed to the formation of a heterojunction between the Fe_2_O_3_ and TiO_2_ nanoparticles that improved charge transfer and suppressed electron–hole recombination. A further investigation on the role of the active species on Fe_2_O_3_/TiO_2_ confirmed that the crucial active species were both holes and superoxide radicals. The Fe_2_O_3_(0.5)/TiO_2_ sample also showed good stability and reusability, suggesting its potential for water purification applications.

## Introduction

Photocatalytic reactions have been widely suggested for environmental remediation under mild conditions. In the presence of only a photocatalyst and a light source of appropriate energy, the process can mineralize organic pollutants to harmless products such as carbon dioxide and water. Among the semiconductor photocatalysts, titanium dioxide (TiO_2_) has been the foremost established material for degradation of organic pollutants [1–2]. In addition to its nontoxicity, abundance and relatively low cost, TiO_2_ also shows excellent photocatalytic activity in many degradation reactions. Unfortunately, the photocatalytic performance of TiO_2_ is generally restricted by its high charge carrier recombination rate. Therefore, the modification of TiO_2_ in order to reduce such recombinations remains a critical task. Another important point is the emphasis on using an environmentally safe and sustainable material as the modifier.

As one of the best modifiers, the use of a co-catalyst has been recognized to improve the photocatalytic performance of semiconductor photocatalysts as it promotes charge separation and suppresses photocorrosion of the semiconductor photocatalyst [3–4]. One of the potential co-catalyst modifiers is iron(III) oxide (Fe_2_O_3_), which is nontoxic, stable, cost effective and found abundantly in the earth. It has been reported that Fe_2_O_3_ can be used to increase the photocatalytic activity or selectivity of semiconductor photocatalysts for degradation of organic pollutants [5–15]. Commonly, the reported methods for the preparation of Fe_2_O_3_/TiO_2_ include impregnation [5–6,16–18], sol–gel [7,19], and hydrothermal methods [8–10]. A combination of several processes has also been employed, such as the electrospinning method combined with a hydrothermal approach [11], plasma enhanced-chemical vapor deposition (PE-CVD) and radio frequency (RF) sputtering approach [12], and plasma enhanced-chemical vapor deposition and atomic layer deposition (ALD) followed by thermal treatment [13]. Among these preparation methods, impregnation is a commonly used approach for the preparation of Fe_2_O_3_/TiO_2_ as it offers a simple process. However, there are contradicting reports on the performance of Fe_2_O_3_/TiO_2_ catalysts prepared by the impregnation method. While some groups reported good photocatalytic activity [5–6], others showed contrasting results [16–18], which have resulted in different opinions regarding the contribution of the Fe_2_O_3_. Since the impregnation method usually involves heat treatment, the properties of TiO_2_ such as the ratio of anatase/rutile, particle size, as well as specific surface area may be altered during this process and could influence the photocatalytic activity of TiO_2_ [16–17]. Therefore, careful considerations shall be taken before concluding whether the Fe_2_O_3_ is beneficial or not in regards to improving the photocatalytic activity of TiO_2_.

Another simple method to produce Fe_2_O_3_/TiO_2_ is a mechano-chemical milling approach that can be carried out at ambient conditions [14]. Even though high activity was obtained, evidence of the formation of good contact between Fe_2_O_3_ and TiO_2_ nanoparticles was not provided. Recently, the photodeposition method has been proposed as a suitable method to directly investigate the role of added copper or lanthanum species without such heat-treatment effects [20–21]. Moreover, the modification of TiO_2_ nanoparticles by photodeposition resulted in an improved photocatalytic activity as compared to unmodified TiO_2_ [20–22]. Therefore, it is meaningful to employ the photodeposition method to prepare Fe_2_O_3_/TiO_2_ catalysts without heat treatment at ambient conditions. Using iron(III) nitrate nonahydrate as the precursor, active and stable Fe_2_O_3_/TiO_2_ was successfully prepared via photodeposition [15]. However, the actual amount of iron precursor in the prepared Fe_2_O_3_/TiO_2_ was much lower than that added. In the present study, Fe_2_O_3_/TiO_2_ nanocomposites were prepared by a similar approach but using iron(III) acetylacetonate as the precursor to facilitate a complete photodeposition process. The properties and activity results were compared with those prepared by the commonly used impregnation approach. Furthermore, to the best of our knowledge, there is no study on the activity comparison between Fe_2_O_3_/TiO_2_ prepared by the widely used impregnation and the photodeposition methods, which is important to determine the optimal method for the preparation of photocatalyst materials with good properties.

In this study, both impregnation and photodeposition methods were used to modify TiO_2_ nanoparticles with Fe_2_O_3_ in order to investigate the effect of preparation method on the properties and photocatalytic activity of the nanocomposites with respect to the degradation of 2,4-dichlorophenoxyacetic acid (2,4-D) under irradiation of UV light. 2,4-D is a herbicide widely utilized in the agricultural industry; it can be found in water sources due to its common use in controlling broadleaf weeds [23]. Excessive exposure of 2,4-D leads to adverse impacts on the ecosystem, and thus, the toxic organic pollutant must be eliminated from the water source utilizing efficient approaches. Various removal methods of 2,4-D have been developed, including adsorption [24], biodegradation [25], ozonation [26], and photocatalytic degradation [15,20–22,27–32], of which the latter has been recognized for its capability to decompose the organic pollutant under a mild environment. In the present work, it was shown that the different preparation methods resulted in distinctly different properties and photocatalytic activity. Better properties and the improved activity of Fe_2_O_3_/TiO_2_ nanocomposites prepared by photodeposition for the degradation of 2,4-D were discussed. In addition to identifying the charge transfer capability of the Fe_2_O_3_/TiO_2_ catalyst for improved photocatalytic activity, the role of the active species on the Fe_2_O_3_/TiO_2_ nanocomposites prepared by the photodeposition method was further investigated in order to understand the important active species contributing to the photocatalytic activity.

## Results and Discussion

### Photocatalytic activity comparison

**Figure 1:**
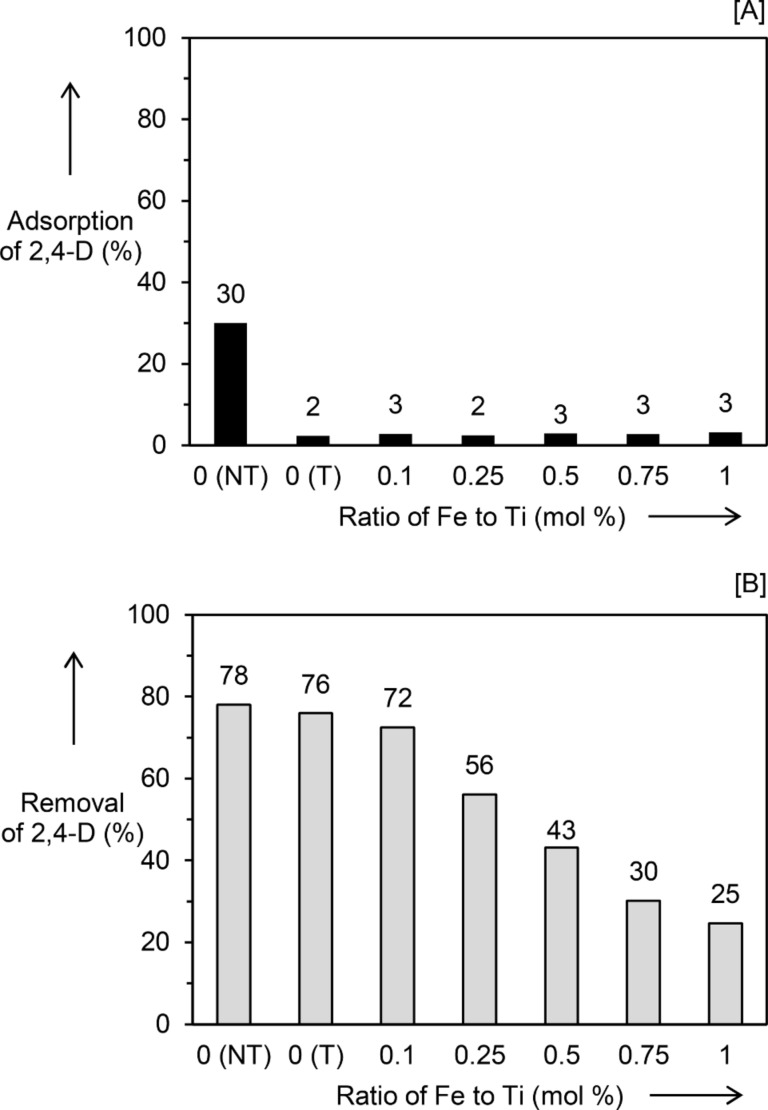
(A) Adsorption and (B) photocatalytic removal of 2,4-D using TiO_2_ (NT), TiO_2_ (IM_T) and series of Fe_2_O_3_/TiO_2_(IM). NT represents no treatment, IM shows the samples were prepared by impregnation method, and T indicates an additional heat treatment was carried out on the sample.

The photocatalytic efficiency of the Fe_2_O_3_/TiO_2_ nanocomposites prepared by impregnation was evaluated for the removal of 2,4-D under UV light illumination at room temperature for 1 h. Under the same conditions, it was confirmed that no photolysis of 2,4-D was obtained without photocatalyst. After adsorption–desorption equilibrium was achieved in 1 h, adsorption experiments were conducted in the absence of light for another 1 h. Related to the following sample descriptions, NT represents no treatment, IM indicates the samples were prepared by impregnation, PD indicates samples were prepared by photodeposition, and T indicates an additional heat treatment was carried out. Figure 1A demonstrates that the TiO_2_ (NT) sample gave 30% adsorption of 2,4-D. After heat treatment at 500 °C, the adsorption of 2,4-D on the samples was greatly suppressed. All the TiO_2_ (IM_T) and Fe_2_O_3_/TiO_2_ (IM) nanocomposites showed 2,4-D adsorption of 2–3%. The photocatalytic activity of the photocatalysts was each determined by exclusion of 2,4-D adsorption and the results are shown in Figure 1B. There was no significant difference observed between the TiO_2_ (NT) and the TiO_2_ (IM_T), which showed 2,4-D removal of 78 and 76%, respectively. Introducing various amounts of Fe_2_O_3_ on the TiO_2_ material via impregnation did not improve the photocatalytic activity of the TiO_2_. With increased loading of Fe_2_O_3_, the photocatalytic performance of TiO_2_ in fact decreased. As another control experiment, α-Fe_2_O_3_ synthesized at 500 °C for 4 h was also tested for the removal of 2,4-D. The removal of 2,4-D using α-Fe_2_O_3_ was only 2% after 1 h of UV illumination, which might be due to the fast charge recombination in hematite [13,15,33].

**Figure 2:**
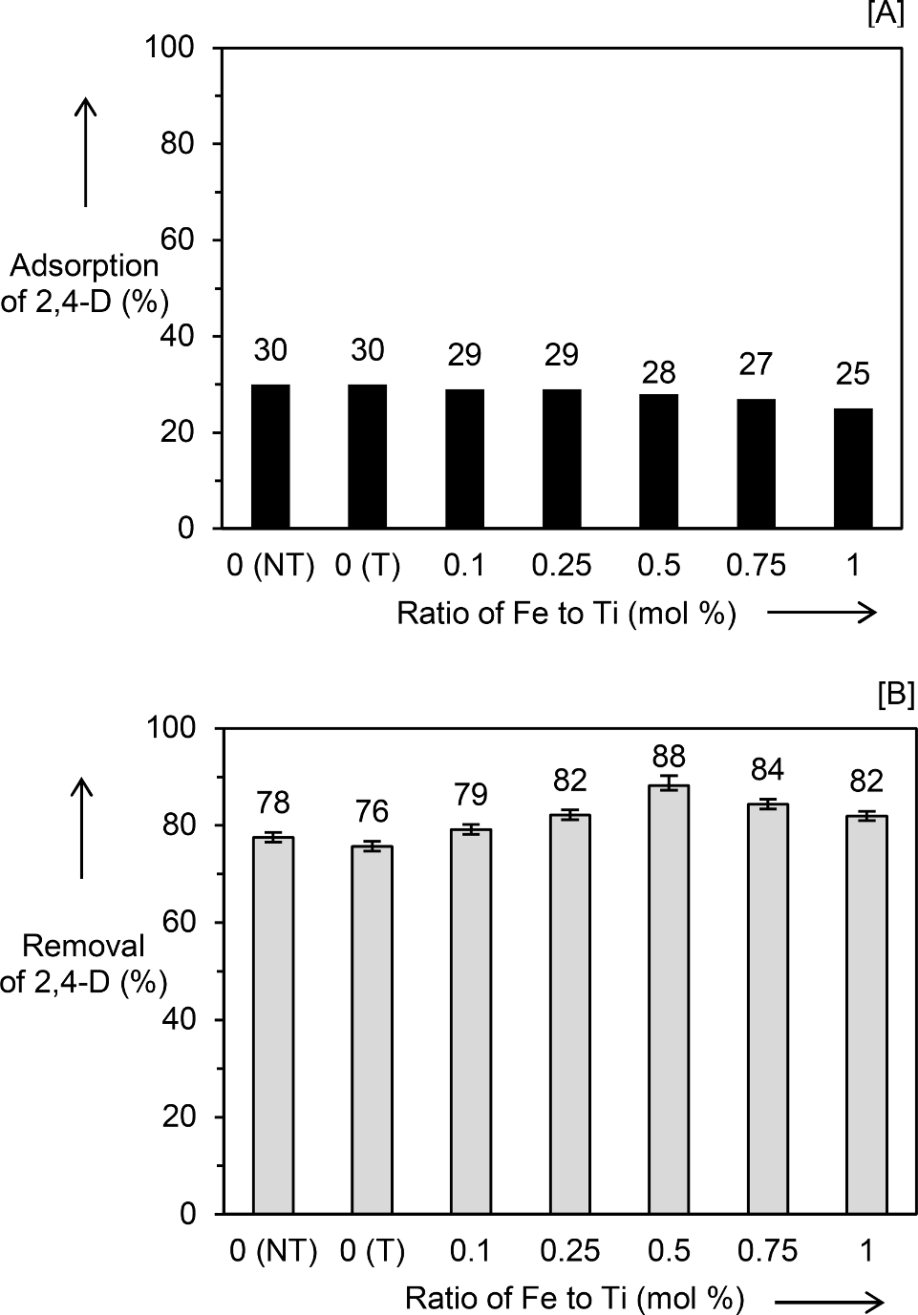
(A) Adsorption and (B) photocatalytic removal of 2,4-D over TiO_2_ (NT), TiO_2_ (PD_T) and a series of Fe_2_O_3_/TiO_2_(PD) samples. Error bars in (B) are shown for comparison purposes. NT represents no treatment, PD shows the samples were prepared by photodeposition method, and T indicates an additional photodeposition treatment was carried out on the sample.

In contrast to the samples synthesized by the impregnation method, the high adsorption of 2,4-D at 25–30% was still achieved on the photodeposition synthesized samples as shown in Figure 2A. Only a slight decrease in adsorption was obtained with increasing Fe/Ti ratio, suggesting that the adsorption sites were not covered by the deposition of Fe_2_O_3_. Figure 2B shows the photocatalytic performance of the TiO_2_ and the Fe_2_O_3_/TiO_2_ (PD) nanocomposites after the exclusion of the 2,4-D adsorption. No significant difference in the activity was obtained for the TiO_2_ (NT) and the TiO_2_ (PD_T), which showed 2,4-D removal of 78 and 76%, respectively. This result clearly demonstrated that, in contrast to the heat treatment, the photodeposition treatment did not alter the photocatalytic performance of TiO_2_. It is worth noting that after the Fe species were photodeposited on the TiO_2_, all the nanocomposites gave superior activity as compared to that of unmodified TiO_2_. The Fe/Ti ratio of 0.5 mol % was found to be the optimum loading in which the Fe_2_O_3_(0.5)/TiO_2_ (PD) sample showed the highest removal of 88% after 1 h irradiation. These results showed that different synthesis methods lead to different photocatalytic performance. The photocatalysts prepared by photodeposition showed superior performance compared to those prepared by the impregnation method.

### Properties comparison

The structural, optical, and physical properties of the Fe_2_O_3_/TiO_2_ photocatalysts synthesized by impregnation and photodeposition were investigated and compared to clarify the characteristic differences of the photocatalysts obtained from the different preparation methods. X-ray diffraction (XRD) patterns were collected for the Fe_2_O_3_/TiO_2_ (IM) series prepared by the impregnation method. TiO_2_ (NT) exhibited diffraction peaks corresponding to the anatase phase (JCPDS file No. 21-1272), which were observed at 2θ of 25.35, 38.10, 48.05, 54.55, and 62.60°, corresponding to (101), (004), (200), (105), and (204) diffraction planes, respectively (see Supporting Information File 1, Figure S1). After heat treatment, the TiO_2_ (IM_T) sample showed improved crystallinity without any changes in the structural phase, which was found to be pure anatase. After addition of Fe species, the crystallinity of the Fe_2_O_3_/TiO_2_ (IM) nanocomposites did not change and was confirmed to be similar to that of the TiO_2_ (IM_T) sample. The characteristic diffraction peaks corresponding to the anatase phase of TiO_2_ remained in all samples without any peak shifting. Furthermore, the existence of new diffraction peaks of α-Fe_2_O_3_ (JCPDS file No. 33-0664) was not identified, suggesting that the low loading of Fe_2_O_3_ might be dispersed well on the surface of the TiO_2_.

The Scherrer equation was used to calculate the crystallite size of the samples based on the (101) peak at 2θ of 25.35°. As listed in Table 1, the crystallite size of the TiO_2_ (NT) was initially 9.3 nm (Table 1, entry 1). After heat treatment, the crystallite size of TiO_2_ (IM_T) increased to 14.3 nm (Table 1, entry 2). The addition of Fe_2_O_3_ did not further influence the crystallite size. All the Fe_2_O_3_/TiO_2_ (IM) nanocomposites had a crystallite size in a range of 14.3–15.9 nm (Table 1, entries 3–7), which was close to that of the TiO_2_ (IM_T). Since there was no much difference in the crystallinity and crystallite size between the TiO_2_ (IM_T) and Fe_2_O_3_/TiO_2_ (IM), it was suggested that the improved crystallinity and crystallite size as compared to TiO_2_ (NT) was mostly due to the heat treatment only and not to the addition of Fe_2_O_3_.

**Table 1:** Crystallite size and band gap energy (*E*_g_) of the unmodified TiO_2_ and Fe_2_O_3_/TiO_2_nanocomposites prepared by impregnation (IM) and photodeposition (PD) methods. NT represents no treatment and T indicates an additional heat treatment was carried out on the sample.

Entry	Samples	Crystallite size (nm)^a^	*E*_g_ (eV)^b^

1	TiO_2_ (NT)	9.3	3.30
2	TiO_2_ (IM_T)	14.3	3.29
3	Fe_2_O_3_(0.1)/TiO_2_ (IM)	14.3	3.29
4	Fe_2_O_3_(0.25)/TiO_2_ (IM)	15.9	3.27
5	Fe_2_O_3_(0.5)/TiO_2_ (IM)	15.8	3.27
6	Fe_2_O_3_(0.75)/TiO_2_ (IM)	15.8	3.26
7	Fe_2_O_3_(1)/TiO_2_ (IM)	15.8	3.25

8	TiO_2_ (PD_T)	8.8	3.29
9	Fe_2_O_3_(0.1)/TiO_2_ (PD)	9.3	3.28
10	Fe_2_O_3_(0.25)/TiO_2_ (PD)	8.8	3.27
11	Fe_2_O_3_(0.5)/TiO_2_ (PD)	8.8	3.27
12	Fe_2_O_3_(0.75)/TiO_2_ (PD)	8.8	3.25
13	Fe_2_O_3_(1)/TiO_2_ (PD)	9.3	3.24

^a^Scherrer equation was used to calculate the crystallite size. ^b^Tauc plot was used to determine the *E*_g_.

The XRD patterns of the Fe_2_O_3_/TiO_2_ (PD) series that was synthesized by the photodeposition method were also recorded (see Supporting Information File 1, Figure S2). Different from the case of heat treatment with the impregnation method, the photodeposition treatment did not change the crystallinity of both the TiO_2_ (PD_T) and the Fe_2_O_3_/TiO_2_ (PD) nanocomposites. No peak shifting and the appearance of no new diffraction peak suggested good dispersion of the Fe species on the surface of the TiO_2_. The crystallite size of the Fe_2_O_3_/TiO_2_ (PD) is given in Table 1. All samples have a crystallite size in the range of 8.8–9.3 nm (Table 1, entries 8–13), suggesting that the crystallite size was not altered by the photodeposition method. Comparing the two synthesis methods, it was obvious that the photodeposition method maintained both crystallinity and crystallite size of the TiO_2_, while the impregnation method led to higher crystallinity and crystallite size. This difference was caused by the different preparation conditions; the photodeposition was conducted under mild synthesis conditions under illumination of UV light at room temperature, whereas a high heating temperature of 500 °C was used during the impregnation method.

The optical absorption properties of the nanocomposites prepared by the impregnation method were investigated (see Supporting Information File 1, Figure S3). The TiO_2_ (NT) sample absorbs light in the UV region and exhibits a characteristic band for TiO_2_ at about 370 nm due to the charge transfer of O^2−^→Ti^4+^ and electron excitation from the valence band (VB) to the conduction band (CB) [7,20–21]. Both the heat treatment and addition of Fe species did not affect the light absorption of the TiO_2_ (NT) in the UV and visible region. Owing to the low loading of Fe, there was no additional absorption peak corresponding to the Fe species. The bandgap energy (*E*_g_) of the unmodified TiO_2_ and the nanocomposites were studied by a Tauc plot, considering the indirect transition in anatase TiO_2_ [34]. The Tauc plot of the TiO_2_ (NT) and the Fe_2_O_3_/TiO_2_ (IM) nanocomposites was derived by plotting (α*h*v)^1/2^ versus *h*v. The *E*_g_ value was obtained from the *x*-intercept using the linear extrapolation in the plot. Table 1 summarizes the *E*_g_ values of the samples. The TiO_2_ (NT) sample has an *E*_g_ of 3.30 eV (Table 1, entry 1). The heat-treated TiO_2_ (IM_T) showed an *E*_g_ value of 2.29 eV (Table 1, entry 2), close to the value of the TiO_2_ (NT), indicating that a high calcination temperature of 500 °C did not affect the optical properties of the TiO_2_. The addition of Fe species did not result in significant changes to the *E*_g_ of the TiO_2_, which with an increasing Fe/Ti ratio from 0.1 to 1 mol % only slightly reduced the *E*_g_ from 3.29 to 3.25 eV (Table 1, entries 3–7). The insignificant change in the *E*_g_ suggested that the Fe species might be loaded on the surface instead of incorporated into the TiO_2_ lattice. The obtained results matched well with the nanocomposite prepared via adsorption and decomposition of the Fe(III) complex at 400 °C [5]. This is in contrast to the one prepared by the sol–gel method that showed an obviously reduced *E*_g_ value as the Fe ions were incorporated into the TiO_2_ lattice [7,19].

Diffuse reflectance (DR) UV–vis spectra and Tauc plots of the nanocomposites prepared by the photodeposition method were also measured (see Supporting Information File 1, Figure S4). Similar to the nanocomposites prepared by the impregnation method, the photodeposition treatment and addition of Fe species also did not much affect the light absorption or the *E*_g_ of both the TiO_2_ (PD_T) and Fe_2_O_3_/TiO_2_ (PD) sample. Besides, the slightly decreased *E*_g_ from 3.28 to 3.24 eV (Table 1, entries 9–13) also suggested that Fe species might be loaded on the surface of the TiO_2_ via photodeposition.

The amount of Fe content loaded on the Fe_2_O_3_/TiO_2_ nanocomposites was determined by the inductively coupled plasma optical emission spectrometer (ICP-OES) measurement, as listed in Table 2. The Fe/Ti composition (mol %) obtained from the measurement confirmed that the Fe content loaded on the TiO_2_ was close to the nominal added amount. These results clearly suggested that in the given range of Fe loading (0.1–1 mol %), all the iron precursor was successfully photodeposited onto the TiO_2_.

**Table 2:** The composition of the Fe_2_O_3_/TiO_2_ (PD) nanocomposites (ratio of Fe/Ti (mol %)) determined from ICP-OES measurements. PD indicates samples that were prepared with the photodeposition method.

Samples	Fe/Ti (mol %)

Fe_2_O_3_(0.1)/TiO_2_ (PD)	0.11
Fe_2_O_3_(0.25)/TiO_2_ (PD)	0.20
Fe_2_O_3_(0.5)/TiO_2_ (PD)	0.45
Fe_2_O_3_(0.75)/TiO_2_ (PD)	0.72
Fe_2_O_3_(1)/TiO_2_ (PD)	1.02

**Figure 3:**
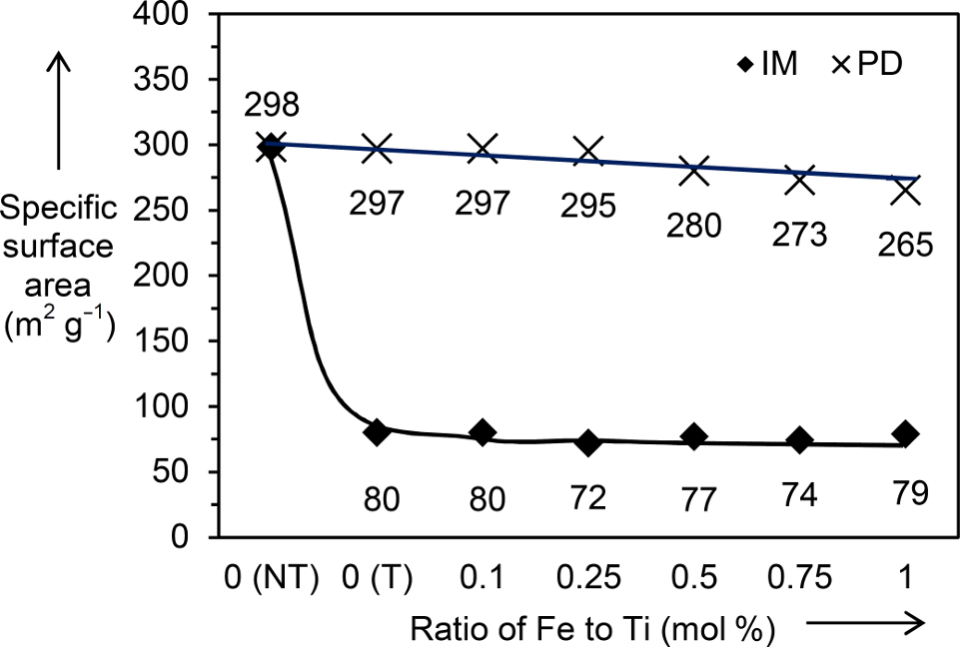
BET specific surface area of TiO_2_ (NT), TiO_2_ (T) and the series of Fe_2_O_3_/TiO_2_ samples prepared by both impregnation (IM) and photodeposition (PD). NT represents no treatment and T indicates an additional heat treatment or photodeposition treatment was carried out on the sample.

The Brunauer–Emmett–Teller (BET) specific surface area of the TiO_2_ and the Fe_2_O_3_/TiO_2_ nanocomposites prepared by the impregnation and the photodeposition methods are shown in Figure 3. The TiO_2_ (NT) has a large specific surface area of 298 m^2^/g. After calcination at 500 °C, the specific surface area of the TiO_2_ (IM_T) dropped drastically to 80 m^2^/g. The addition of Fe_2_O_3_ to TiO_2_ via the impregnation method did not significantly change the specific surface area of the TiO_2_ (IM_T), given that all nanocomposites have values in the range of 72–80 m^2^/g. This result obviously showed that it was the heat treatment and not the Fe_2_O_3_ addition that caused the decrease in the BET specific surface area.

In contrast to the nanocomposites prepared by the impregnation method, only a slight gradual decrease was observed with increasing Fe/Ti ratio in the Fe_2_O_3_/TiO_2_ nanocomposites prepared from the photodeposition method. The nanocomposite sample with the lowest Fe/Ti ratio of 0.1 mol % still showed a large surface area of 297 m^2^/g, while the nanocomposite sample with the highest Fe/Ti ratio of 1 mol % showed a value of 265 m^2^/g. These results again confirmed that the mild photodeposition method did not influence the properties of the TiO_2_ (NT).

As shown in Figure 1 and Figure 2, nanocomposites synthesized by the photodeposition method exhibited superior adsorption and photocatalytic activity than those synthesized by the impregnation method. The higher percentage of 2,4-D adsorption could result from the larger BET specific surface area of the samples prepared by the photodeposition method. As for the photocatalytic activity, a few important parameters have been reported to contribute to a high photocatalytic activity, including high crystallinity [35], small crystallite size [36], and high specific surface area [30,36]. Generally, materials with high crystallinity have fewer crystal defects, while a smaller crystallite size decreases the diffusion path length between the charge carriers − these two parameters can suppress recombination of photogenerated electrons−holes. On the other hand, materials with a large specific surface area have many available surface active sites for reaction to take place, which can lead to high photocatalytic activity. In the case of nanocomposites prepared by the impregnation method, even though improved crystallinity was observed, it might be compensated by the larger crystallite size and a lower specific surface area, which overall led to decreased photocatalytic activity. Since the photodeposition method did not have a great influence on the crystallinity, crystallite size, and the BET specific surface area, the effects caused by such changes can be avoided, and the main factors contributing to the photocatalytic activity can be narrowed down solely to the added Fe species.

### Improved properties

**Figure 4:**
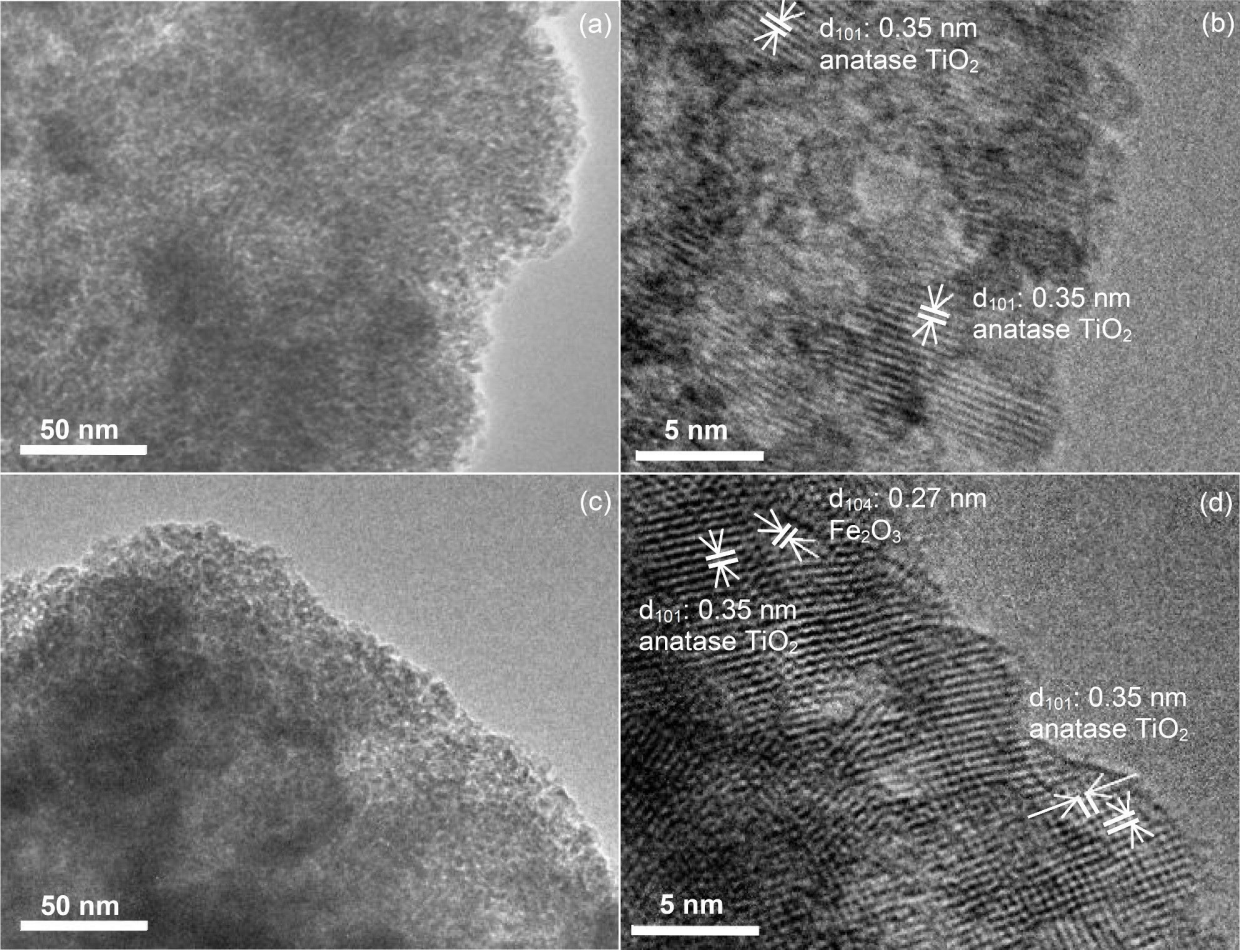
(a) TEM image of unmodified TiO_2_ (NT) and (b) its respective HRTEM image, (c) TEM image of Fe_2_O_3_(0.5)/TiO_2_ (PD) and (d) its respective HRTEM image.

Since nanocomposites synthesized by the photodeposition method showed better photocatalytic activity than the nanocomposites synthesized by the impregnation method, further detailed investigations were carried out on nanocomposites synthesized by the photodeposition method. Transmission electron microscopy (TEM) and high-resolution TEM (HRTEM) images of both unmodified TiO_2_ (NT) and Fe_2_O_3_(0.5)/TiO_2_ (PD) are shown in Figure 4. As shown in Figure 4a, the TiO_2_ (NT) sample has spherical particles with a diameter of 7–9 nm. This result agreed well with the crystallite size calculated by the Scherrer equation previously discussed. The HRTEM image of the TiO_2_ (NT) sample displayed in Figure 4b shows a lattice fringe spacing of 0.35 nm attributed to the anatase TiO_2_(101) crystal plane. Figure 4d shows a HRTEM image of Fe_2_O_3_(0.5)/TiO_2_ (PD). It was evident that the deposition of Fe did not change the morphology of the TiO_2_. Since the lattice fringe spacing of 0.27 nm related to the Fe_2_O_3_(104) crystal plane was observed, the possible formation of a heterojunction between Fe_2_O_3_ and TiO_2_ was considered. Such close contact would cause the carrier diffusion length to be short, and in turn, would improve the charge transfer. This would thus suppress charge recombination, which is crucial to enhance the photocatalytic activity.

The formation of Fe_2_O_3_ was in good agreement with other reported photodeposition methods when using a different iron precursor, Fe(III) nitrate nonahydrate [15]. Due to the oxidative condition during the synthesis process, the Fe(III) acetylacetonate precursor could be decomposed to Fe_2_O_3_ such as by the photogenerated oxygen radicals [21]. It was demonstrated that the use of the Fe(III) acetylacetonate precursor led to a complete photodeposition to form Fe_2_O_3_, as also supported by ICP-OES results discussed above.

**Figure 5:**
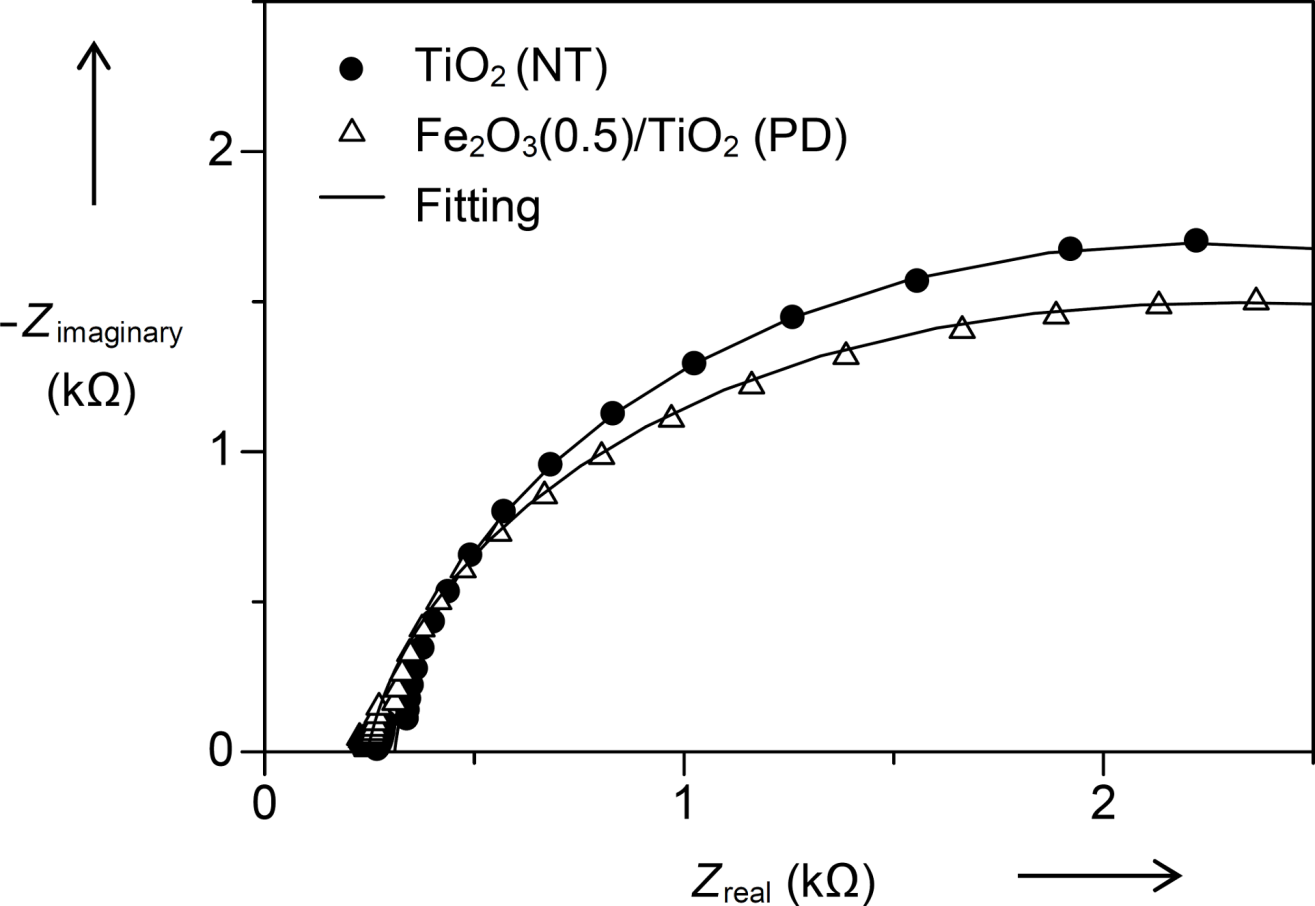
Nyquist plots of unmodified TiO_2_ (NT) and Fe_2_O_3_(0.5)/TiO_2_ (PD) with the respective model fitting.

The improved charge transfer of the Fe_2_O_3_(0.5)/TiO_2_ (PD) sample was further clarified using electrochemical impedance spectroscopy (EIS). Figure 5 shows the Nyquist plots of the unmodified TiO_2_ (NT) and Fe_2_O_3_(0.5)/TiO_2_ (PD) samples. The arc radius of the Nyquist plot reflects the impedance of the interface layer arising at the electrode surface. The smaller the arc radius the better the charge transfer [37]. It is worth noting here that the Fe_2_O_3_(0.5)/TiO_2_ (PD) material has a smaller arc radius than unmodified TiO_2_. These results clearly suggest that the Fe_2_O_3_(0.5)/TiO_2_ (PD) material has a lower impedance than unmodified TiO_2_, indicating enhanced conductivity of TiO_2_ after photodeposition of Fe_2_O_3_. The electron transfer kinetics of a material can be calculated using Equation 1:

[1]
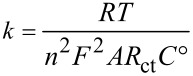


where *k* is the heterogeneous electron-transfer rate constant, *R* is the gas constant, *T* is temperature (K), *n* represents the number of transferred electrons per molecule of the redox probe, *F* is the Faraday constant, *A* is the electrode area (cm^2^), *R*_ct_ is the charge transfer resistance that can be obtained from the fitted Nyquist plot, and *C*° is the concentration of the redox couple in the bulk solution (ferricyanide/ferrocyanide) [38].

From the fitted impedance data shown in Figure 5, the Fe_2_O_3_(0.5)/TiO_2_ (PD) material gave an *R*_ct_ value of 2.87 kΩ, which was smaller than that of unmodified TiO_2_ (NT) with *R*_ct_ = 3.40 kΩ. The lower *R*_ct_ value obviously suggested that the Fe_2_O_3_(0.5)/TiO_2_ (PD) material has better charge conductivity and transfer capability than unmodified TiO_2_ (NT). Furthermore, the *k* values of the Fe_2_O_3_(0.5)/TiO_2_ (PD) sample and unmodified TiO_2_ (NT) were calculated to be 2.96 × 10^−4^ and 2.49 × 10^−4^ cm/s, respectively, indicating that the charge transfer that on the Fe_2_O_3_(0.5)/TiO_2_ (PD) sample proceeded faster than on unmodified TiO_2_. As suggested from the HRTEM result, the formation of an Fe_2_O_3_/TiO_2_ heterojunction might promote better electron transfer which resulted in improved photocatalytic activity of the Fe_2_O_3_(0.5)/TiO_2_ (PD) material.

**Figure 6:**
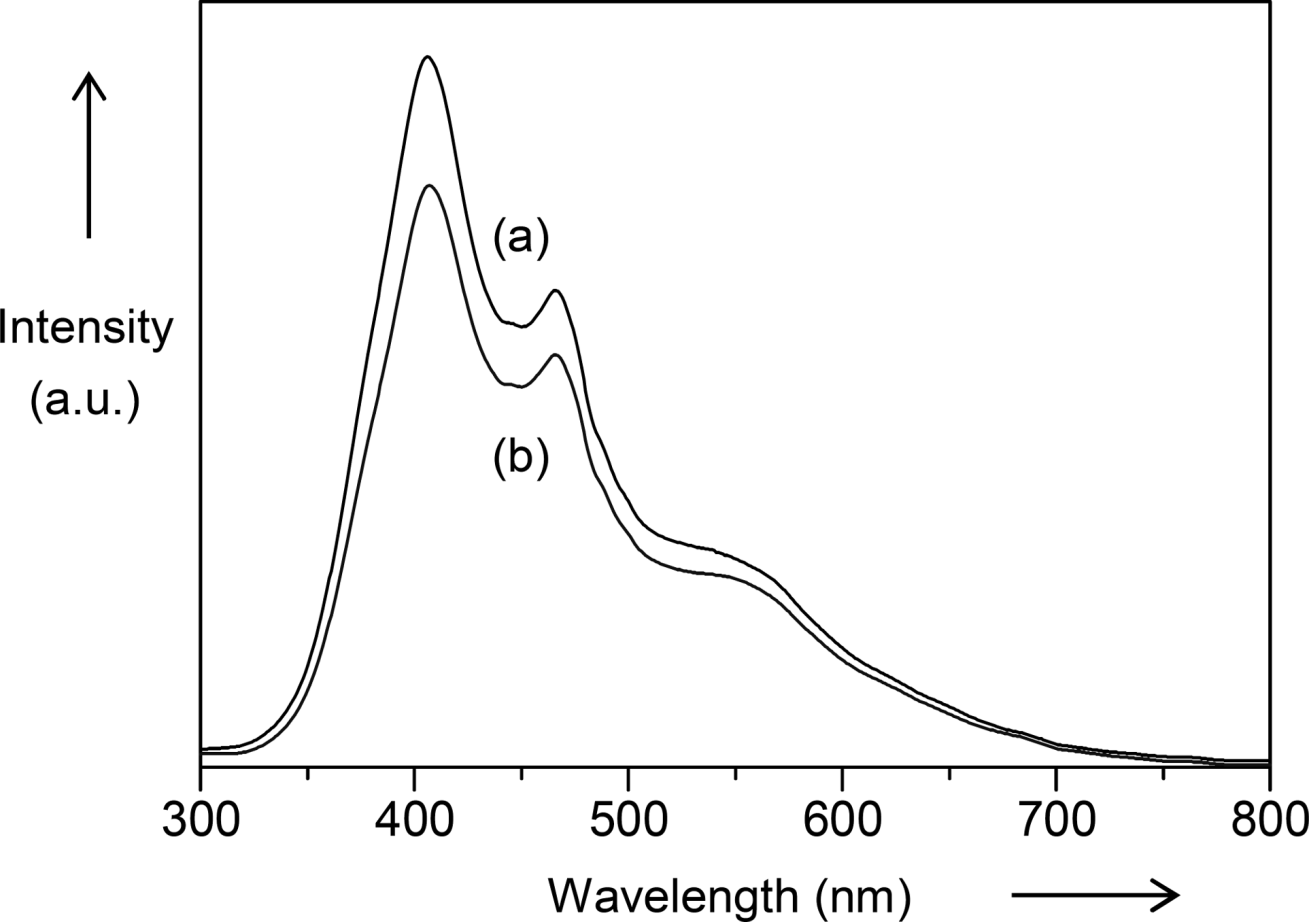
Emission spectra of (a) unmodified TiO_2_ (NT) and (b) Fe_2_O_3_(0.5)/TiO_2_ (PD).

Photoluminescence has been associated with electron–hole recombination of a photocatalyst [39]. In this study, the ability of an Fe_2_O_3_ co-catalyst to accept photogenerated electrons as well as to suppress the recombination of electron–holes on the TiO_2_ was supported by the fluorescence spectroscopy results. The emission spectra of the unmodified TiO_2_ (NT) and the Fe_2_O_3_(0.5)/TiO_2_ (PD) samples under a fixed excitation wavelength of 218 nm are shown in Figure 6. TiO_2_ exhibited three emission peaks at 407, 466 and 562 nm. The emission at 407 nm could be caused by the radiative recombination of self-trapped excitons, while peaks at 466 and 562 nm were attributed to the charge transfer of an oxygen vacancy trapped electron. The obtained results agreed well with the reported literature [39]. The Fe_2_O_3_(0.5)/TiO_2_ (PD) material showed a decreased emission intensity as compared to the unmodified TiO_2_ (NT), suggesting that the photogenerated electrons on TiO_2_ could be transferred and trapped by Fe_2_O_3_. This resulted in a suppression of the electrons−hole recombination on TiO_2_, which led to the improved removal of 2,4-D.

For comparison, a Nyquist plot and emission spectrum of the Fe_2_O_3_(0.1)/TiO_2_ (IM) material were also measured and given in Supporting Information File 1, Figures S5 and S6, respectively. It was clear that the Fe_2_O_3_(0.1)/TiO_2_ (IM) had a smaller arc radius of the Nyquist plot and slightly lower emission intensity than the TiO_2_ (NT), suggesting that the Fe_2_O_3_(0.1)/TiO_2_ (IM) has better charge transfer and suppressed electron–hole recombination. Unfortunately, these better properties did not promote the photocatalytic activity of the Fe_2_O_3_(0.1)/TiO_2_ (IM). It turns out that the photocatalytic activity of Fe_2_O_3_(0.1)/TiO_2_ (IM) would be more influenced by the distinct decrease in the specific surface area, as discussed previously.

### Active species and stability

**Figure 7:**
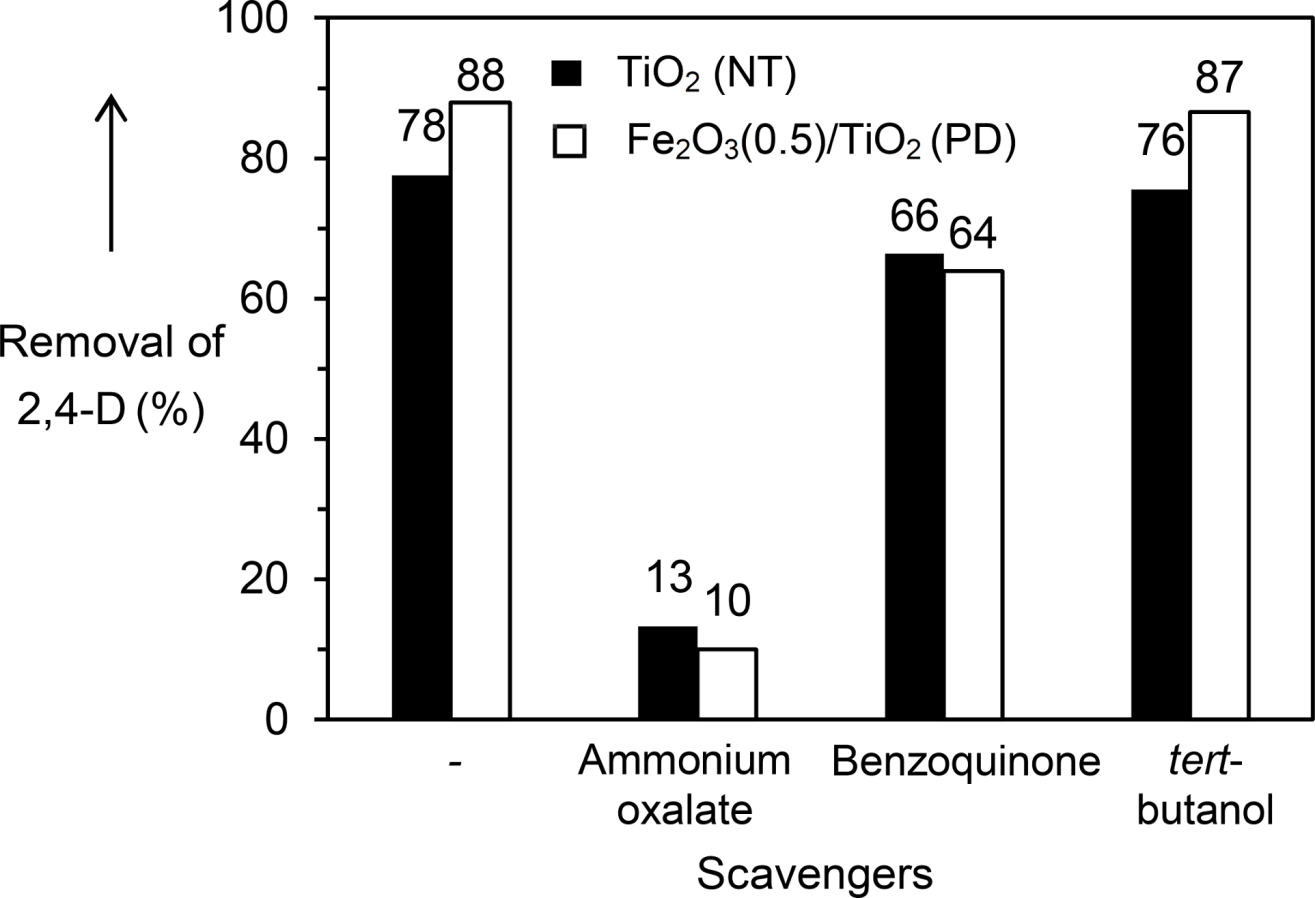
Percentage removal of 2,4-D on unmodified TiO_2_ (NT) and Fe_2_O_3_(0.5)/TiO_2_ (PD) in the absence and presence of various scavengers under UV light irradiation for 1 h.

It has been reported that the reaction pathways for photocatalytic oxidation of organic pollutants are dominated by several active species, such as holes, superoxide radicals, and hydroxyl radicals [39]. Among the scavengers of active species, ammonium oxalate has been reported as an efficient hole scavenger [40], benzoquinone acts to scavenge superoxide radicals efficiently [40], while *tert*-butanol has fast reaction with hydroxyl radicals [27,40] and hence, they were selected for the scavenger studies. As shown in Figure 7, the photocatalytic reactions under 1 h of UV illumination were evaluated in the presence of each scavenger on the unmodified TiO_2_ (NT) and the Fe_2_O_3_(0.5)/TiO_2_ (PD). For the reaction conducted on the unmodified TiO_2_ (NT), the addition of ammonium oxalate was found to drastically suppress the activity, which was reduced from 78 to 13%, equivalent to 5.8 times lower than the one without scavenger. The inhibited activity indicated the importance of the photogenerated holes for the oxidation of 2,4-D. When benzoquinone was added, the activity was reduced from 78 to 66%, suggesting that superoxide radicals also played a role in the oxidation process of 2,4-D. In contrast, addition of *tert*-butanol did not affect the activity of the TiO_2_ (NT), indicating that hydroxyl radicals are not the important active species for the reaction.

Since the photogenerated holes on the TiO_2_ have strong oxidizing power among oxidizing species [41], it is reasonable that holes are the most dominate active species in the oxidation of 2,4-D. Moreover, it has been reported that the oxidation of 2,4-D via a direct holes mechanism was favored at pH 3 [27]. In this study, an initial pH for 2,4-D was confirmed to be 3.2. On the other hand, superoxide radicals could be also easily formed for the oxidation reaction since the reaction was conducted in an open reactor, whereby the reduction of oxygen can easily take place. Related to the formation of hydroxyl radicals, it has been revealed that more hydroxyl radicals are formed from the adsorbed hydroxide ions with increased pH [28,42]. Therefore, it is likely that under the present conditions, they did not contribute as the active species probably due to their low formation.

The scavenger study was also conducted using the Fe_2_O_3_(0.5)/TiO_2_ (PD) as shown in Figure 7. It was clear that the Fe_2_O_3_(0.5)/TiO_2_ (PD) gave similar trend of activity as the ones obtained on the unmodified TiO_2_ (NT). Both the photogenerated holes and superoxide radicals were important species, while hydroxyl radicals did not give much influence on the photocatalytic oxidation of 2,4-D. As compared to the unmodified TiO_2_ (NT), the Fe_2_O_3_(0.5)/TiO_2_ (PD) showed a more drastic reduction in the activity when the reactions were conducted in the presence of holes and superoxide radical scavengers. The activity decreased 8.8 and 1.4 times, respectively, as compared to those on TiO_2_ (NT), i.e., 5.8 and 1.2 times, respectively. Such a result suggested the crucial role of Fe_2_O_3_ as a co-catalyst to improve the interfacial charge transfer and suppress electron–hole recombination. This leads to the formation of more photogenerated holes and superoxide radicals that contributed to an improved photocatalytic activity, as was also supported by the HRTEM, EIS and fluorescence spectroscopy results.

The stability of the Fe_2_O_3_(0.5)/TiO_2_ (PD) sample was investigated by performing several cycles of photocatalytic reactions under UV light irradiation for 1 h. The Fe_2_O_3_(0.5)/TiO_2_ (PD) sample gave a similar, comparable activity in a range of 82–88% even after 3 cycles of reactions, suggesting the good photostability of the Fe_2_O_3_(0.5)/TiO_2_ (PD) nanocomposite and its potential application for photocatalytic water purification.

### Degradation and proposed mechanism

**Figure 8:**
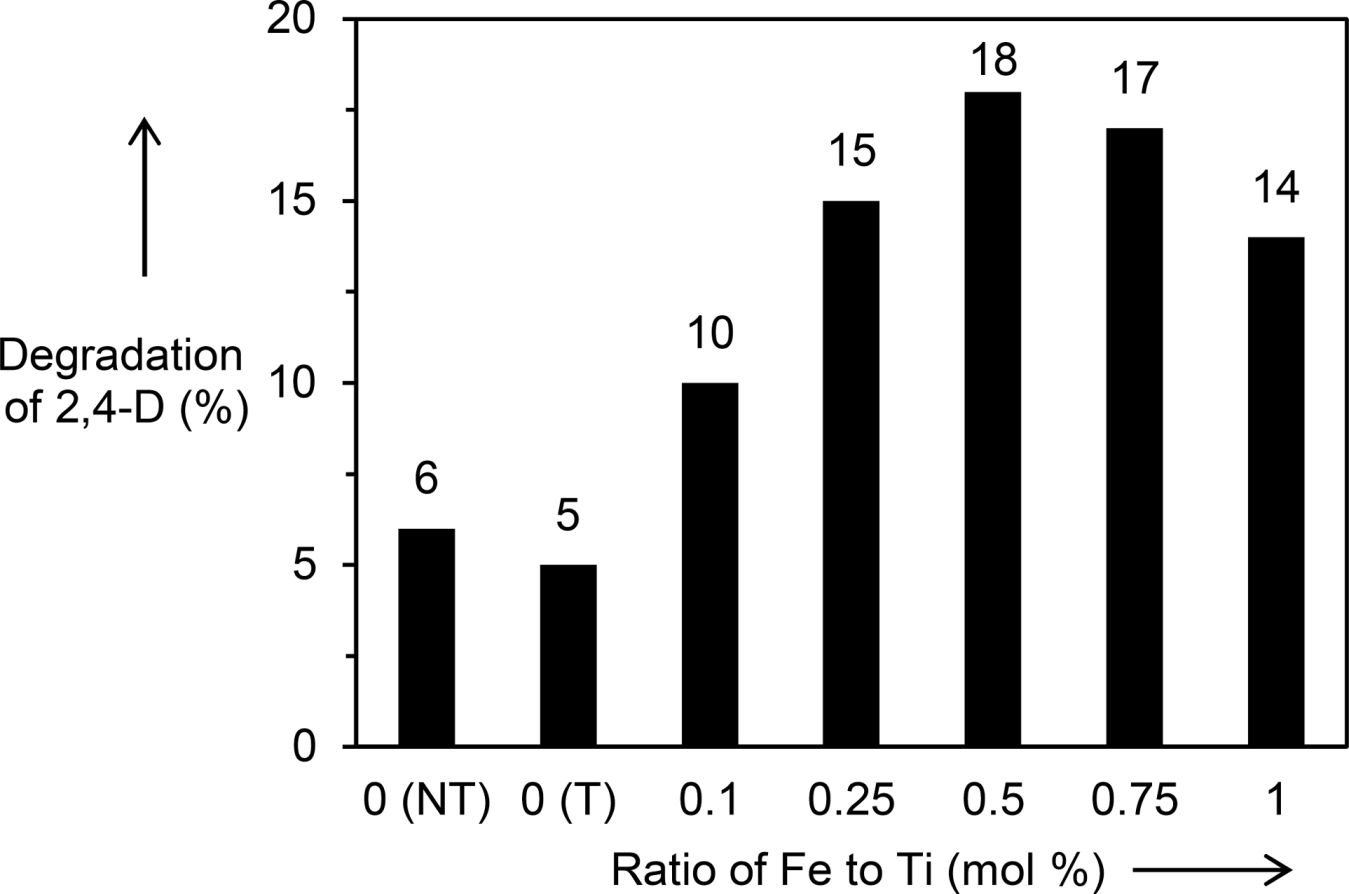
Photocatalytic degradation of 2,4-D on TiO_2_ (NT), TiO_2_ (PD_T) and the series of Fe_2_O_3_/TiO_2_(PD) samples. NT represents no treatment, PD shows the samples were prepared by photodeposition method, and T indicates an additional photodeposition treatment was carried out on the sample.

After the photocatalytic reactions on all samples, the formation of a 2,4-dichlorophenol (2,4-DCP) intermediate was observed from the HPLC analysis, which was in good agreement with reported studies [15,28–32]. The 2,4-D degradation was then determined by Equation 2:

[2]
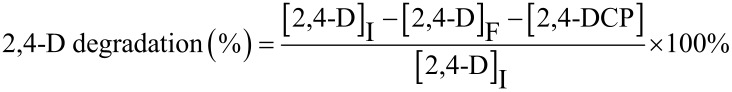


where [2,4-D]_I_ represents the initial concentration of 2,4-D after reaching adsorption–desorption equilibrium under dark conditions, [2,4-D]_F_ is the final concentration of 2,4-D after lamp exposure and [2,4-DCP] is the concentration of the formed 2,4-DCP intermediate after lamp exposure. The percentage of 2,4-D degradation on the unmodified TiO_2_ and the Fe_2_O_3_/TiO_2_ (PD) series is given in Figure 8. Unmodified TiO_2_ (NT) and TiO_2_ (PD_T) showed a comparable degradation of 2,4-D of 6 and 5%, respectively. The addition of Fe_2_O_3_ was demonstrated to improve the photocatalytic activity of TiO_2_ for degradation of 2,4-D. The Fe_2_O_3_(0.5)/TiO_2_(PD) showed a 2,4-D degradation of 18%, which was three times higher than the unmodified TiO_2_ (NT). Such enhanced performance was only slightly higher than that reported when using a Fe(III) nitrate nonahydrate precursor, which gave more than two times higher activity than the bare TiO_2_ [15].

The photocatalytic oxidation of 2,4-D by active species involves various steps, including formation of intermediates before its mineralization to CO_2_ and H_2_O. Decarboxylation has been reported as the initial step during the photocatalytic oxidation of 2,4-D when it is carried out at pH 3 [27]. The benzene ring opening and hydrocarbon chain breaking then took place, which finally led to the formation of CO_2_ [29]. Since 2,4-DCP was detected as the dominant intermediate after the photocatalytic reactions, it could be suggested that 2,4-D was firstly oxidized by the active species (photogenerated holes and superoxide radicals) before decarboxylation and the formation of 2,4-DCP. The dechlorination of 2,4-DCP then took place, leading to ring opening, hydrocarbon chain breaking, and finally, the mineralization to CO_2_ and H_2_O (see Supporting Information File 1, Figure S7).

**Figure 9:**
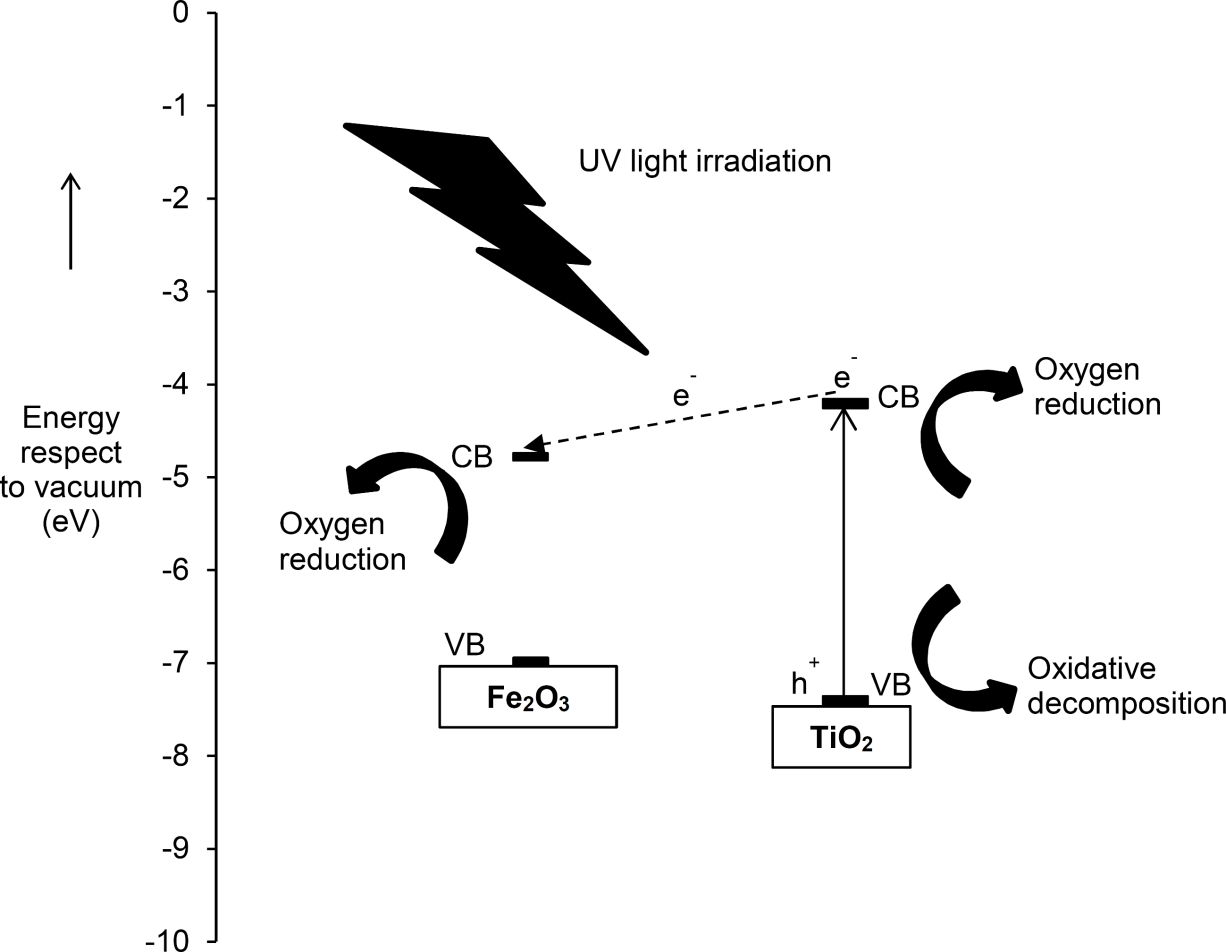
Proposed mechanism for major charge transfer pathways on Fe_2_O_3_(0.5)/TiO_2_ (PD) for degradation of 2,4-D.

The mechanism of major charge transfer pathways on the Fe_2_O_3_(0.5)/TiO_2_ (PD) was also proposed and shown in Figure 9. When the photocatalyst is exposed to UV light, photogenerated electrons are excited from the VB to the CB of TiO_2_, while photogenerated holes are left in the VB. The photogenerated electrons could reduce oxygen to form superoxide radicals, while holes could directly oxidize 2,4-D to 2,4-DCP before its mineralization. The presence of Fe_2_O_3_ reduces electron–hole recombination on the TiO_2_. Since the CB edge energy level of Fe_2_O_3_ (−4.78 eV relative to absolute vacuum scale (AVS)) is lower than that of TiO_2_ (−4.21 eV relative to AVS) [43], Fe_2_O_3_ could act as an electron trapper that captured the photogenerated electrons from the TiO_2_ that were not used for reduction of oxygen, instead of recombination with holes. Such electron transfer could suppress charge recombination on TiO_2_ [5,10,12,14–15], whereby the oxidation of 2,4-D still could occur in the VB of TiO_2_, and therefore, the photocatalytic degradation of 2,4-D could be improved. On the other hand, owing to the fast recombination of holes and electrons, the photocatalytic degradation of 2,4-D on bare Fe_2_O_3_ was negligible (1%). The oxidation of 2,4-D is unlikely to take place in the valence band of Fe_2_O_3_ and this would be the very minor pathway. Similar mechanisms have been also reported elsewhere [15].

## Conclusion

Two series of Fe_2_O_3_/TiO_2_ nanocomposites were prepared by the impregnation and the photodeposition methods. The Fe_2_O_3_/TiO_2_ nanocomposites prepared by the impregnation method showed less activity than the unmodified TiO_2_ (NT), which was mainly due to the lower specific surface area caused by heat treatment. On the other hand, all the Fe_2_O_3_/TiO_2_ nanocomposites prepared by the photodeposition methods exhibited superior photocatalytic activity as compared to the unmodified samples. The good photocatalytic activity of the nanocomposites was associated with the formation of a heterojunction between Fe_2_O_3_ and TiO_2_ nanoparticles that promoted good charge transfer and suppressed electron–hole recombination. Scavenger studies showed that the photogenerated holes and superoxide radicals were the important active species in the reaction. The Fe_2_O_3_(0.5)/TiO_2_ material showed excellent stability and reusability for the removal of 2,4-D. Among the nanocomposites, the Fe_2_O_3_(0.5)/TiO_2_ sample showed the best activity, exhibiting 18% degradation of 2,4-D after 1 h of reaction, corresponding to three times higher activity compared to unmodified TiO_2_.

## Experimental

### Materials

All chemicals and materials in the experiments were used without supplementary purification. The chemicals used were commercial Hombikat UV100 TiO_2_ (UV100, Sachtleben Chemie), iron(III) acetylacetonate (99.9%, Sigma–Aldrich), ethanol (99.98%, HmbG^®^ Chemicals), sodium sulfate (99.0%, Fisher Chemical), potassium ferricyanide (99.0%, Riedel-de Haën), 2,4-D (98.0%, Sigma), ammonium oxalate (99.5–101%, Merck), benzoquinone (99%, Acros Organics), and *tert*-butanol (99.0%, Merck).

### Sample preparation

The TiO_2_ material used in this study was from the commercial supplier Hombikat, UV100 TiO_2_. The Fe_2_O_3_ used as a control was prepared by direct calcination of Fe(III) acetylacetonate under air atmosphere at 500 °C for 4 h. Two series of Fe_2_O_3_/TiO_2_ nanocomposites were prepared by impregnation and photodeposition methods. As for the synthesis of the nanocomposites via the impregnation method, an appropriate amount of Fe(III) acetylacetonate with varying mole percentage (mol %) of Fe/Ti of 0.1, 0.25, 0.5, 0.75 and 1 mol % were firstly dissolved in mixed solvents of water and ethanol (20 mL). Then, the commercial Hombikat UV100 TiO_2_ (1 g) was dispersed in the Fe(III) acetylacetonate solution for 10 min by an ultrasonicator. The mixture was stirred and heated at 80 °C until all solvents were completely evaporated. The grind dried solid powder was then calcined at a temperature of 500 °C for 4 h. The prepared samples were labelled as Fe_2_O_3_(x)/TiO_2_ (IM), where x relates to the loading of Fe/Ti in mol %. Bare TiO_2_ with a similar heat treatment without the addition of the iron precursor was also prepared and denoted as TiO_2_ (IM_T), while the TiO_2_ without any pretreatment was denoted as TiO_2_ (NT).

As for synthesis of the nanocomposites via the photodeposition method [20–22], an appropriate amount of Fe(III) acetylacetonate with various mole percentages of Fe/Ti (0.1, 0.25, 0.5, 0.75 and 1 mol %) were firstly dissolved in mixed solvents of water and ethanol (20 mL) by ultrasonication for few minutes. Then, the commercial Hombikat UV100 TiO_2_ (1 g) was dispersed in the Fe(III) acetylacetonate solution by ultrasonic mixing for 10 min. The mixture was then stirred and irradiated under a 200 W Hg−Xe lamp (Hamamatsu, light intensity of 8 mW/cm^2^ at 365 nm) at room temperature for 5 h. The solid was washed a few times with ethanol followed by deionized water before drying overnight inside an oven at 80 °C. Finally, the obtained solid powder was ground. The prepared samples were denoted as Fe_2_O_3_(x)/TiO_2_ (PD), where x relates to the loading of Fe/Ti (in mol %). Bare TiO_2_ undergoing a similar photodeposition treatment without the addition of the iron precursor was also produced and was denoted as TiO_2_ (PD_T).

### Sample characterization

A Bruker D8 Advance diffractometer was used to measure the XRD patterns of the TiO_2_ and the Fe_2_O_3_/TiO_2_ samples prepared by both impregnation and photodeposition methods using a Cu Kα radiation source (λ = 0.15406 nm) at 40 kV and 40 mA. A Shimadzu UV-2600 DR UV−vis spectrophotometer was used to record the absorption spectra of samples, in which barium sulfate (BaSO_4_) was used as a reference. The elemental compositions (Fe, Ti) on the Fe_2_O_3_/TiO_2_ (PD) nanocomposites were determined using an Agilent 700 series ICP-OES. The adsorption of nitrogen gas on the samples was measured at 77 K on a Quantachrome Novatouch LX4 instrument in order to determine the BET specific surface area of the samples.

TEM and HRTEM were performed on a JEOL JEM-2100 electron microscope with electron acceleration energy of 200 kV. EIS measurements were performed on a Gamry Interface 1000 potentiostat/galvanostat/ZRA. For the measurements of EIS, a screen printed electrode (SPE, DropSens) was used and prepared as follows. The photocatalyst sample (10 mg) was dispersed in water (6 mL) and the mixture was homogeneously mixed in an ultrasonic bath for 15 min. The mixture (20 µL) was then dropped onto the working electrode of the SPE, followed by immersion of the SPE in 6 mL of electrolyte which was a mixture of sodium sulfate (0.1 M) and potassium ferricyanide (2.5 mM). The frequency range was set in the range of 1 MHz to 100 mHz. A simplex model program (Gamry Echem Analyst) was selected to fit the obtained Nyquist plot by using constant phase element (CPE) with diffusion as the equivalent circuit model. The emission sites of the samples were investigated using a JASCO FP-8500 spectrofluorometer, in which the excitation wavelength was fixed at 218 nm. The reproducibility for emission spectra measurements was around 4%.

### Photocatalytic tests

The photocatalytic activity of the Fe_2_O_3_/TiO_2_ nanocomposites prepared by both photodeposition and impregnation methods was tested for the removal of 2,4-D under irradiation of UV light for 1 h. The photocatalyst (50 mg) was dispersed in a 2,4-D solution (0.5 mM, 50 mL) and stirred for 1 h in the dark to achieve adsorption–desorption equilibrium. The photocatalytic reaction was then conducted under irradiation of a 200 W Hg-Xe lamp (Hamamatsu, light intensity of 8 mW/cm^2^ at 365 nm) for 1 h at room temperature. After each reaction, the solution was separated from the photocatalyst by using a membrane filter. The concentration of 2,4-D was determined using a high-performance liquid chromatography instrument (Shimadzu, Prominence LC-20A with Hypersil gold PFP column), which was monitored at a wavelength of 283 nm. The percentage of 2,4-D removal was determined following Equation 3:

[3]
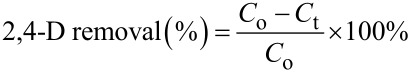


where *C*_o_ is the initial concentration of 2,4-D after reaching adsorption–desorption equilibrium under dark conditions, while *C*_t_ is the remaining concentration of 2,4-D after the reaction. Further investigation on the role of active species contributing to the removal of 2,4-D was carried out on the Fe_2_O_3_(0.5)/TiO_2_ (PD) nanocomposite, which showed the best photocatalytic activity. Ammonium oxalate, benzoquinone, and *tert*-butanol were used as the various scavengers for photogenerated holes, superoxide radicals and hydroxyl radicals, respectively. The scavenger was introduced to the 2,4-D solution in the presence of the photocatalyst with 1 mole ratio of scavenger/pollutant.

The photostability of the Fe_2_O_3_(0.5)/TiO_2_ (PD) nanocomposite was investigated by evaluating the photocatalytic activity for removal of 2,4-D over three cycles. After the first run of reaction under 1 h UV irradiation, the photocatalyst was collected from the 2,4-D solution and was washed with deionised water before drying at 80 °C overnight. The second and third cycles of reactions were conducted using the recovered photocatalyst under similar experimental and treatment conditions, as mentioned above.

## Supporting Information

File 1 Additional figures.
The supporting information file contains seven figures with additional experimental data labelled as Figure S1–S7.
