# Supplementary material for: High photocatalytic activity of Fe2O3/TiO2 nanocomposites prepared by photodeposition for degradation of 2,4-dichlorophenoxyacetic acid
Source: Beilstein J Nanotechnol. 2017 Apr 24;8:915–26. doi: 10.3762/bjnano.8.93 (PMC5433238; doi:10.3762/bjnano.8.93)
Supplement: File 1 — Additional figures. The supporting information file contains seven figures with additional experimental data labelled as Figure S1–S7. [file Beilstein_J_Nanotechnol-08-915-s001.pdf]

Supporting Information
for

High photocatalytic activity of Fe_2O_3/TiO_2 nanocomposites prepared by photodeposition for degradation of 2,4-dichlorophenoxyacetic acid

Shu Chin Lee[1], Hendrik O. Lintang[1,2] and Leny Yuliati*[1,2]

Address: [1]Centre for Sustainable Nanomaterials, Ibnu Sina Institute for Scientific and Industrial Research, Universiti Teknologi Malaysia, 81310 UTM Johor Bahru, Johor, Malaysia and [2]Ma Chung Research Center for Photosynthetic Pigments, Universitas Ma Chung, Villa Puncak Tidar N-01, Malang 65151, East Java, Indonesia

Email: Leny Yuliati - leny.yuliati@machung.ac.id

*Corresponding Author

Additional Figures

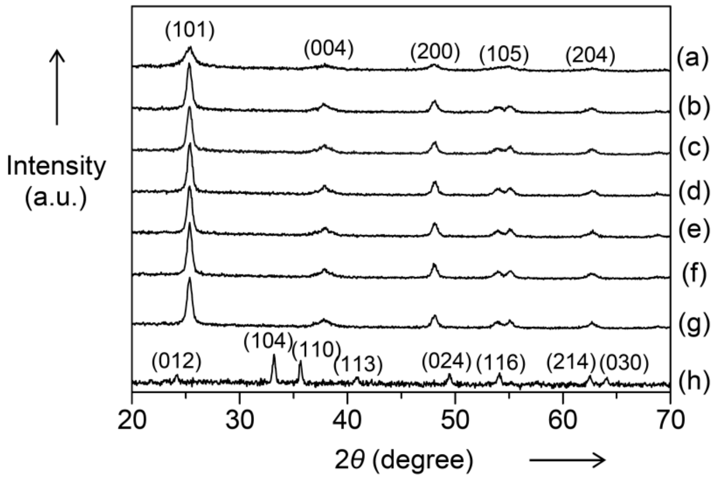

Figure S1: XRD patterns of (a) TiO_2 (NT), (b) TiO_2 (IM_T), (c) Fe_2O_3(0.1)/TiO_2 (IM), (d) Fe_2O_3(0.25)/TiO_2 (IM), (e) Fe_2O_3(0.5)/TiO_2 (IM), (f) Fe_2O_3(0.75)/TiO_2 (IM), (g) Fe_2O_3(1)/TiO_2 (IM), and (h) Fe_2O_3.

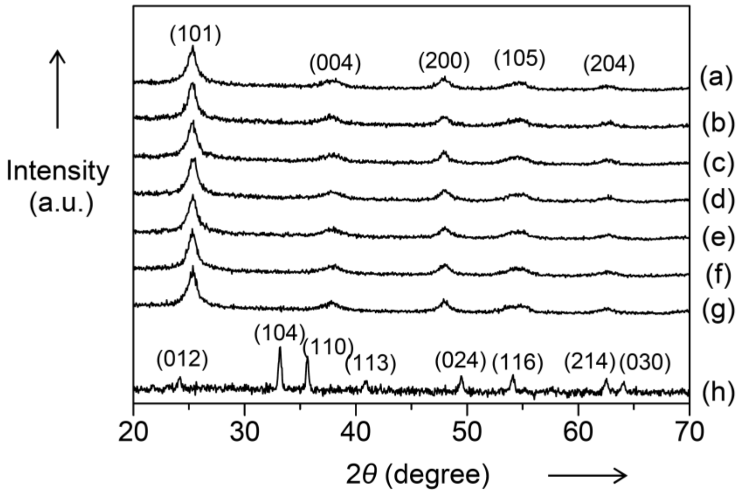

Figure S2: XRD patterns of (a) TiO_2 (NT), (b) TiO_2 (PD_T), (c) Fe_2O_3(0.1)/TiO_2 (PD), (d) Fe_2O_3(0.25)/TiO_2 (PD), (e) Fe_2O_3(0.5)/TiO_2 (PD), (f) Fe_2O_3(0.75)/TiO_2 (PD), (g) Fe_2O_3(1)/TiO_2 (PD), and (h) Fe_2O_3.

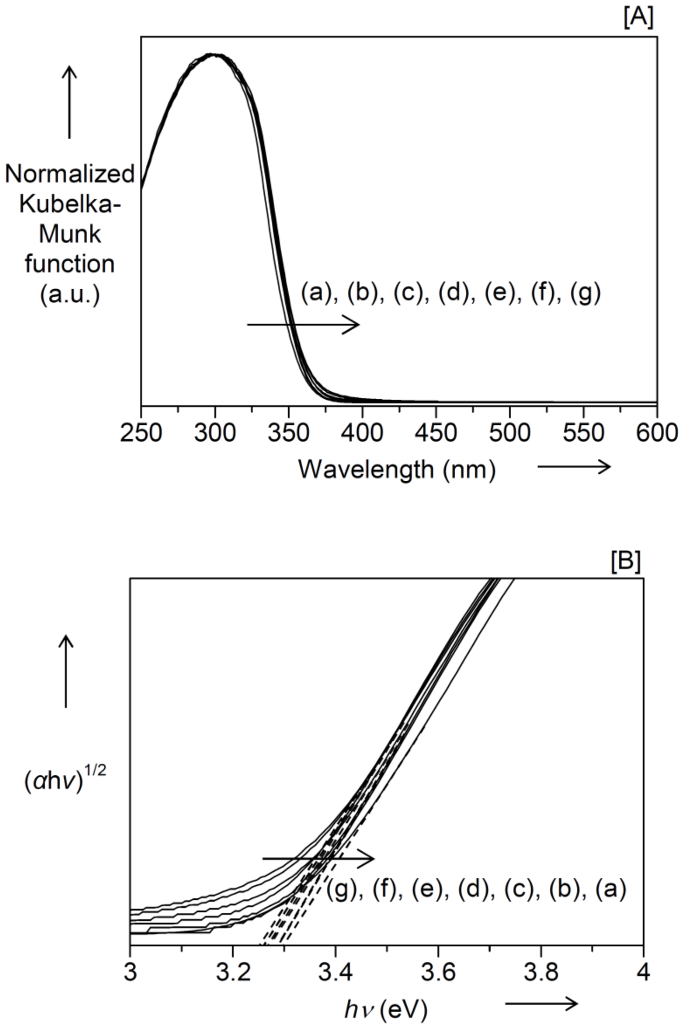

Figure S3: [A] DR UV-visible spectra and [B] Tauc plots of (a) TiO_2 (NT), (b) TiO_2 (IM_T), (c) Fe_2O_3(0.1)/TiO_2 (IM), (d) Fe_2O_3(0.25)/TiO_2 (IM), (e) Fe_2O_3(0.5)/TiO_2 (IM), (f) Fe_2O_3(0.75)/TiO_2 (IM), and (g) Fe_2O_3(1)/TiO_2 (IM).

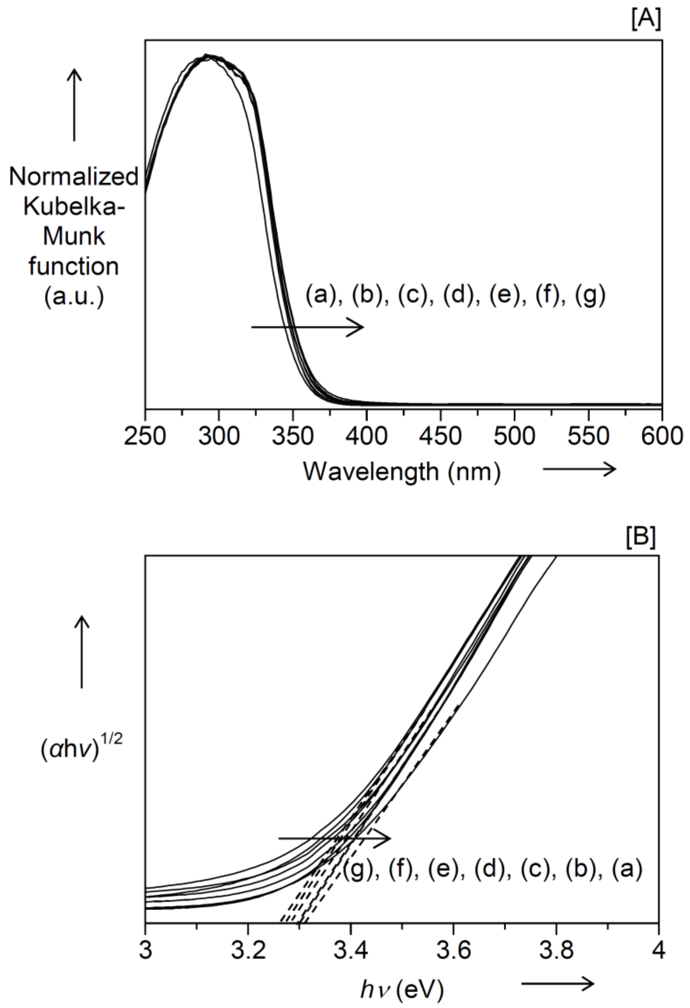

Figure S4: [A] DR UV-visible spectra and [B] Tauc plots of (a) TiO_2 (NT), (b) TiO_2 (PD_T), (c) Fe_2O_3(0.1)/TiO_2 (PD), (d) Fe_2O_3(0.25)/TiO_2 (PD), (e) Fe_2O_3(0.5)/TiO_2 (PD), (f) Fe_2O_3(0.75)/TiO_2 (PD), and (g) Fe_2O_3(1)/TiO_2 (PD).

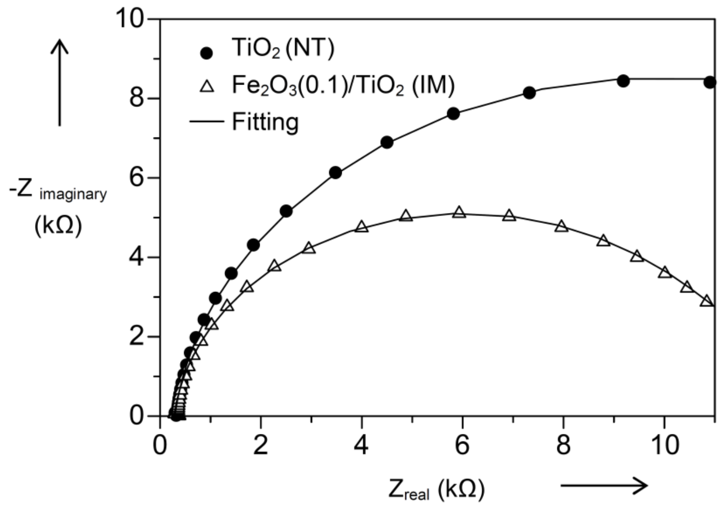

Figure S5: Nyquist plots of unmodified TiO_2 (NT) and Fe_2O_3(0.1)/TiO_2 (IM) with the respective model fitting. 10 mg photocatalyst [TiO_2 (NT) and Fe_2O_3(0.1)/TiO_2 (IM)] and 10 µL nafion (Aldrich, 5%) were dispersed in 1 mL water and were sonicated for 10 min to form a homogeneous colloidal suspension, prior to deposition onto the SPE.

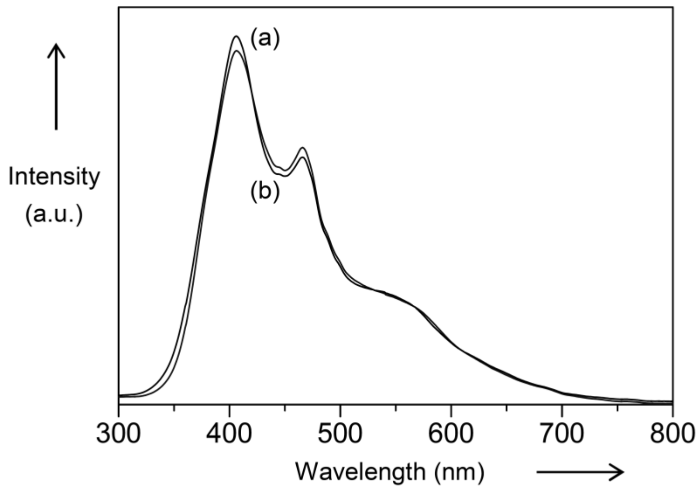

Figure S6: Emission spectra of (a) unmodified TiO_2 (NT) and (b) Fe_2O_3(0.1)/TiO_2 (IM).

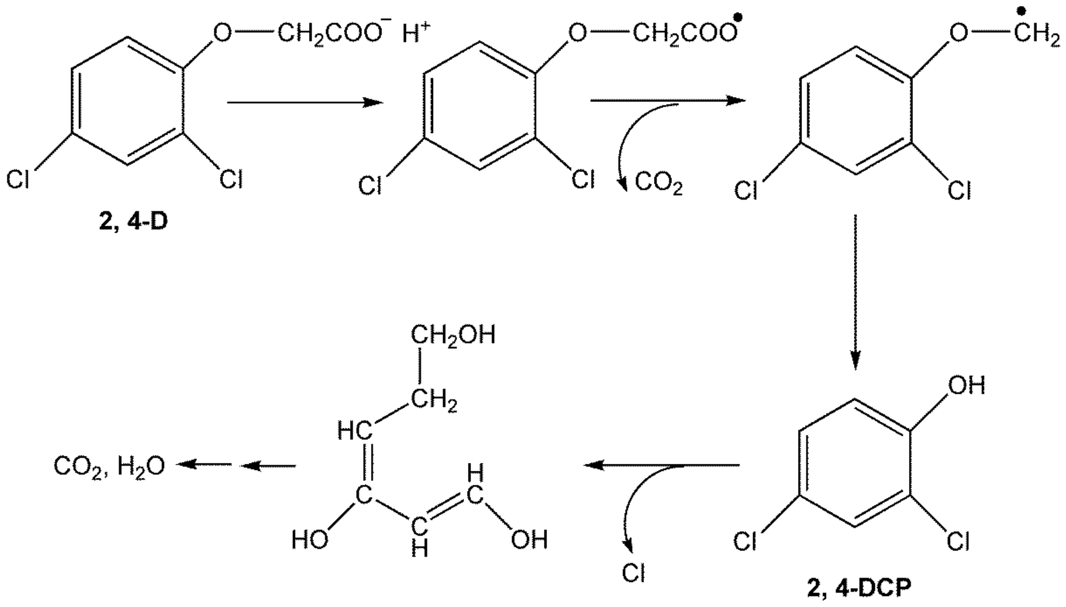

Figure S7: Proposed mechanism for photocatalytic degradation of 2,4-D.
